# Stress-Related Amygdala Metabolic Activity Is Associated With Low Bone Mineral Density in Postmenopausal Women: A Pilot ^18^F-FDG PET/CT Study

**DOI:** 10.3389/fendo.2021.719265

**Published:** 2021-08-12

**Authors:** Kisoo Pahk, Hyun Woo Kwon, Chanmin Joung, Sungeun Kim

**Affiliations:** ^1^Department of Nuclear Medicine, Korea University Anam Hospital, Seoul, South Korea; ^2^Department of Neuroscience, Korea University College of Medicine, Seoul, South Korea

**Keywords:** osteoporosis, psychological stress, amygdala, postmenopause, F-18 FDG PET/CT, PET/CT

## Abstract

**Background:**

Psychological stress is associated with postmenopausal osteoporosis. However, the underlying mechanism of stress-related brain neural activity with osteoporosis is not fully elucidated. ^18^F-fluorodeoxyglucose positron emission tomography/computed tomography (^18^F-FDG PET/CT) is an established method to evaluate the metabolic activity of brain amygdala, a region involved in stress. We aimed to evaluate the relationship between metabolic activity of amygdala (AmygA) and osteoporosis in postmenopausal women.

**Materials and Methods:**

A total of 115 postmenopausal women who underwent ^18^F-FDG PET/CT and dual-energy X-ray absorptiometry for routine health screening were enrolled in this study. AmygA was defined as the maximum standardized uptake value (SUVmax) of amygdala divided by the mean SUV of temporal lobe. The levels of psychological stress were measured using the Psychosocial Well-being Index-Short Form (PWI-SF).

**Results:**

The participants with osteoporosis exhibited significantly higher AmygA than without osteoporosis (0.81 ± 0.16 *vs*. 0.61 ± 0.13, *p* < 0.001). The AmygA value of 0.69 was suggested as an optimal cut-off value to identify participant with osteoporosis (sensitivity; 79.1%, specificity; 83.3%, area under the curve; 0.841, *p* < 0.001). Furthermore, AmygA showed significant association with osteoporosis in postmenopausal woman by multivariate analysis. Psychological stress scale (PWI-SF) was well correlated with AmygA and AmygA was highest in high stress risk-, intermediate in moderate stress risk-, and lowest in healthy group.

**Conclusions:**

AmygA measured by ^18^F-FDG PET/CT is associated with osteoporosis in postmenopausal women. Our results provide the possibility that stress-related neurobiological activity involving amygdala is linked with postmenopausal osteoporosis.

## Introduction

Osteoporosis is a common metabolic bone disease, particularly postmenopausal women, characterized by low bone mineral density (BMD) and deterioration of micro-architectural bone tissue thereby reducing bone mass and strength, which collectively lead to increased susceptibility to fracture (1, 2). World health organization (WHO) has defined osteoporosis by low BMD (T-score ≤ -2.5) and there are approximately 40 million women with low BMD in the United States (1, 2). Globally, increased incidence of osteoporotic fracture further imposes a significant clinical and public health burden (1, 2)

Psychological stress is the emotional and physiological response to social, environmental, and psychological stressors (3). Accumulating evidences have suggested that psychological stress is associated with low BMD and can be a risk factor for osteoporosis in postmenopausal women (4, 5). Psychological stress response proceeds to activate hypothalamic-pituitary-adrenal (HPA) axis thereby increasing the levels of glucocorticoid in systemic circulation (6). Increased glucocorticoid production can suppress the differentiation and proliferation of osteoblast, which is a key cell for maintaining the bone homeostasis (6). Although the physiological stress is known to deteriorate bone structure through the modulation of stress hormone signaling (6), the underlying mechanism between stress-related brain neural activity and osteoporosis is still remained unclear.

Amygdala is a key component of the brain’s salience network, which regulates autonomic, hormonal, and behavioral response to psychological stress (7). Recently, several previous studies have reported that the metabolic activity of amygdala (AmygA) can be reproducibly evaluated using ^18^F-fluorodeoxyglucose positron emission tomography/computed tomography (^18^F-FDG PET/CT) by showing cellular glycolysis, and is associated with individual’s level of psychological stress (8–10). Furthermore, increased AmygA evaluated by ^18^F-FDG PET/CT has been associated with future major adverse cardiovascular events and atherosclerotic plaque vulnerability, for which psychological stress is a significant risk factor (8, 9). Thus, we hypothesized that elevated AmygA could also be related with bone loss and might provide the mechanistic insights into stress-related brain neural activity and osteoporosis in postmenopausal women.

In this study, we aimed to investigate whether the AmygA assessed by ^18^F-FDG PET/CT is associated with osteoporosis and the levels of psychological stress in postmenopausal women.

## Materials and Methods

### Study Population

**Figure 1:**
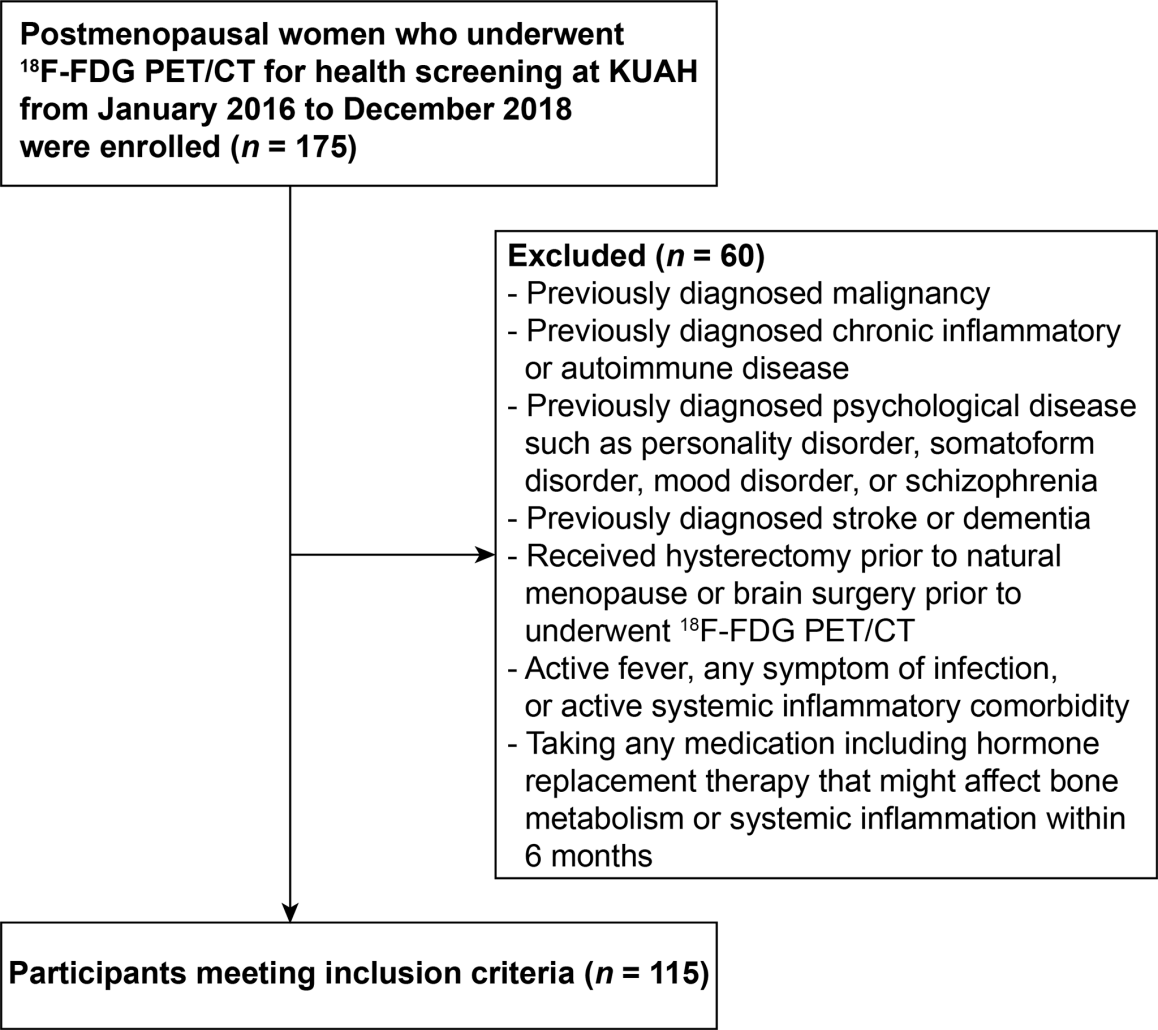
The flowchart of study design. ^18^F-FDG PET/CT, ^18^F-fluorodeoxyglucose positron emission tomography/computed tomography; KUAH, Korea University Anam Hospital.

Participants were identified from a pool of postmenopausal women who underwent ^18^F-FDG PET/CT for health screening at Korea University Anam Hospital Health Promotion Center from January 2016 to December 2018. The study flow chart is shown in **Figure 1**. Participants who received brain surgery prior to underwent ^18^F-FDG PET/CT, or hysterectomy before menopause, who had active systemic inflammatory comorbidity, any symptom of infection, or active fever, or women taking any medication including hormone replacement therapy that might affect systemic inflammation or bone metabolism within 6 months were excluded. Participants who had previously diagnosed malignancy, chronic inflammatory, or autoimmune disease, or psychological disease such as personality disorder, somatoform disorder, mood disorder, or schizophrenia were excluded. In addition, women who had history of stroke or dementia, which could influence physical activity thereby confounding the findings of this study, were also excluded. Finally, a total of 115 participants were included in this study. The Institutional Review Board of Korea University Anam Hospital approved the study design (Approval No. 2019AN0130) and formal consent was waived for this retrospective study.

### Lifestyle-, Anthropometric-, and Laboratory Variables

A fully experienced staff performed a questionnaire to all participants to obtain clinical information such as menopausal age, medical history, the status of smoking and alcohol consumption, and exercise habit. For the evaluation of frequency of alcohol use, “Light” was defined as the participants who drink 2-3 times a week, whereas “Heavy” was defined as the participants who drink greater than 3 times a week. For the evaluation of amount of alcohol use, “Light” was defined as the participants who drink 1 to 3 glasses per occasion, whereas “Heavy” was defined as the participants who drink more than 3 glasses per occasion. One glass was defined as containing 8 g of alcohol, which equates to 200 ml of beer with 4.5% alcohol. For the assessment of exercise activity, “Regular exercise” was defined as the participants who take regular exercise 3 times a week. Exercise was defined as performing more than 30 min of aerobic exercise with moderate-intensity (between 3 and 6 metabolic equivalents) and muscle-resistant exercise using the major muscle groups with more than 8 repetitions in one day. Body mass index (BMI) was defined as weight/height squared (kg/m^2^) and waist circumference (WC) was measured using a flexible tape at the level of the umbilicus between the lower rib and iliac crest. The high-sensitivity C-reactive protein (hsCRP) levels were determined by using Dade Behring BNII analyzer (Siemens, Munich, Germany).

### Psychosocial Well-Being Index-Short Form

The levels of psychological stress were measured using the PWI-SF. PWI-SF is wildly used in Korea and is consisted of 18 items in four-point Likert scale with a total range from 0 to 54 points (11, 12). According to the summed total score, participants were classified as “healthy group” (≤ 8 points), “moderate stress risk group” (9–26 points), and “high stress risk group” (≥ 27 points) (11, 12).

### Measurement of Areal BMD

Areal BMD of the femoral neck (FN) and the lumbar spine (LS) were measured using dual-energy X-ray absorptiometry (Discovery-W, Hologic, Bedford, MA, USA). T-scores were determined from the northeastern Asian young women data (13). The diagnosis of osteoporosis was defined by the T-score ≤ -2.5 at either FN or LS according to the WHO criteria (14).

### ^18^F-18 FDG PET/CT Imaging Acquisition Protocol

All participants received 5.29 MBq/kg (0.14 mCi/kg) ^18^F-FDG intravenously, after at least 6 h fasting to maintain serum glucose level under 180 mg/dL. The PET/CT scan was initiated 1 h after ^18^F-FDG administration using a dedicated PET/CT scanner (Gemini TF, Philips Medical Systems, Cleveland, OH, USA). Imaging acquisition was performed under an unstimulated condition to capture the resting status of neurobiological brain PET signal activities. Non-contrast-enhanced, non-gated CT (120 kVp, 50 mA, and 4 mm slice thickness) was done for attenuation correction followed by three-dimensional (3D) PET scan from the skull vertex to the proximal thigh. Acquired images were reconstructed by iterative algorithm using the 3D-ordered subsets expectation maximization reconstruction algorithm (3 iterations with 33 subsets).

### Imaging Analysis

Images were analyzed by two experienced nuclear medicine physicians (KP and HWK) with more than 10 years of expertise who were blinded to the clinical information of enrolled participants. All analyses were performed using a dedicated workstation (Extended Brilliance Workspace version 3.5, Philips Healthcare, Eindhoven, Netherlands) which enables comprehensive evaluation using standardized uptake value (SUV). SUV was defined as follow: *SUV = Tracer activity (region of interest; ROI) (MBq/mL)/Injected dose (MBq)/Total body weight (g).*

The amygdalae, which are the part of the limbic system located dorsally in the medial temporal lobe, were identified by anatomical landmarks used in previous studies (8–10). In detail, the anterior boundary was defined by the inferior part of the lateral ventricles which were flattened by the thalamus, and the posterior boundary was drawn over the crux of fornix which was anterior to the basilar artery. The lateral and inferior boundaries were defined by the internal capsule. For the assessment of AmygA, a circular ROI (radius < 15mm) was located on each right and left amygdala, as previously described (8–10). Averaged maximum SUVs from those ROIs were obtained and defined as Amyg SUVmax. AmygA was defined as the Amyg SUVmax divided by the mean SUV of temporal lobe which was corrected for background cerebral activity, as previously described (8–10). The intra- and interobserver correlation coefficients of measured SUVs from targeted ROIs were > 0.9.

### Statistical Analysis

All data are expressed as mean ± standard deviation. Normal distribution of data was determined using Shapiro-Wilk test. Continuous variables were analyzed by Student’s *t*-test or Mann-Whitney *U* test and categorical variables were analyzed by chi-squared (χ^2^) test or Fisher’s exact test. One-way analysis of variance (ANOVA) was used for multiple comparisons followed by a *post-hoc* Tukey’s test. Correlations between two variables were analyzed by partial correlation analysis and Spearman’s correlation coefficient. Receiver-operating characteristic (ROC) curve analysis, univariate-, and multivariate logistic regression analysis were also used for statistical analyses. All data analysis was performed using SPSS software version 17.0 (SPSS Inc, Chicago, IL, USA) and MedCalc software version 18.1 (MedCalc, Mariakerke, Belgium). The value of statistical power was set at 0.8 and the statistical significance was determined as a two-tailed *p*-value of < 0.05.

## Results

### Baseline Clinical Characteristics of Enrolled Postmenopausal Women

Among the enrolled 115 participants, 43 women were diagnosed with osteoporosis. Detailed baseline characteristics of enrolled participants are presented in **Table 1**. The osteoporosis group showed significantly higher values for BMI, WC, postmenopausal period, and hsCRP than the participants without osteoporosis. Furthermore, the osteoporosis group had more stress, as assessed by the PWI-SF score than the women without osteoporosis.

**Table 1:** Baseline clinical characteristics of participants.

	Osteoporosis (–)	Osteoporosis (+)	*p*
No. of subjects	72	43	
Age (years)	58.8 ± 6.7	60.4 ± 4.6	0.016*
Height (cm)	157.7 ± 5.1	158 ± 4.3	0.86
Weight (kg)	56.8 ± 9.3	61.5 ± 7.5	<0.001*
BMI (kg/m^2^)	23.1 ± 3.6	24.1 ± 2.7	0.006*
WC (cm)	76.5 ± 7.7	79.6 ± 7.1	0.018*
LS BMD T-score	-0.9 ± 1	-2.7 ± 0.3	<0.001*
FN BMD T-score	-1 ± 0.9	-2.1 ± 1	<0.001*
PWI-SF score	10.3 ± 7.9	17.7 ± 10.1	<0.001*
Postmenopausal period (years)	7 ± 6.2	9.6 ± 4.9	0.002*
Smoking, n (%)			0.498
Never	65 (90.3)	40 (93)	
Ex	5 (6.9)	1 (2.3)	
Current	2 (2.8)	2 (4.7)	
Alcohol frequency, n (%)			0.256
None	40 (55.6)	30 (69.8)	
Light	27 (37.5)	12 (27.9)	
Heavy	5 (6.9)	1 (2.3)	
Alcohol amount, n (%)			0.345
None	40 (55.6)	29 (67.4)	
Light	28 (38.9)	11 (25.6)	
Heavy	4 (5.5)	3 (7)	
Regular exercise, n (%)	13 (18.1)	7 (16.3)	0.808
hsCRP, mg/dL	1.2 ± 1.8	1.8 ± 2.4	0.037*

BMI, body mass index; WC, waist circumference; LS BMD, lumbar spine bone mineral density; FN BMD, femoral neck bone mineral density; PWI-SF, psychosocial well-being index-short form; hsCRP, high-sensitivity C-reactive protein.

*Statistically significant difference.

Age, Weight, BMI, WC, LS BMD T-score, FN BMD T-score, PWI-SF score, Postmenopausal period, and hsCRP were analyzed with Mann-Whitney U test. Height was analyzed with Student’s t-test.

### AmygA Is Increased in Postmenopausal Women With Osteoporosis

**Figure 2:**
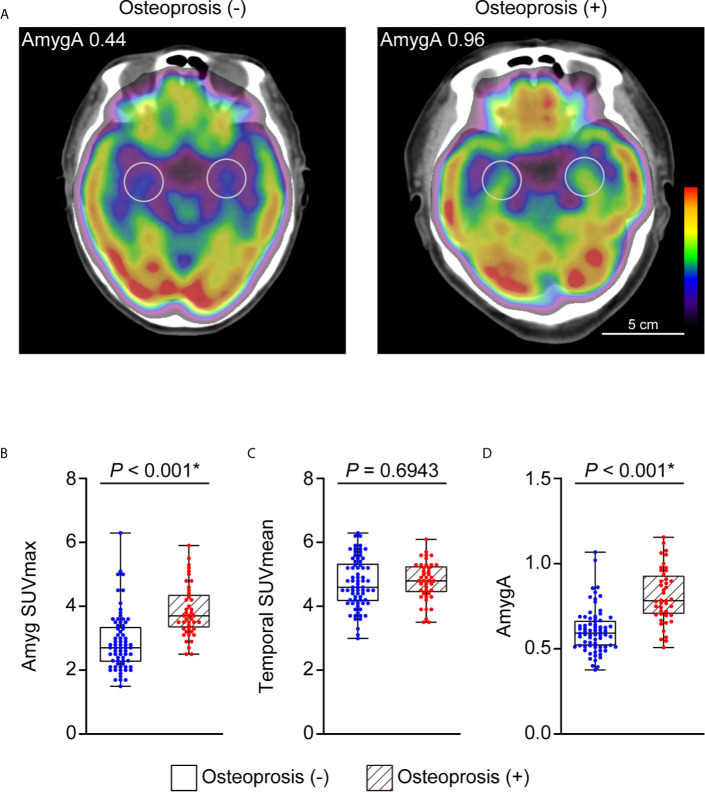
Representative images of AmygA **(A)** and comparison of Amyg SUVmax **(B)**, Temporal SUVmean **(C)**, and AmygA **(D)** between the postmenopausal women diagnosed with and without osteoporosis. Osteoporosis (-), *n* = 72; Osteoporosis (+), *n* = 43. Amyg SUVmax; maximum standardized uptake value of amygdala, Temporal SUVmean; mean standardized uptake value of temporal lobe, AmygA; the metabolic activity of amygdala; Amyg SUVmax/Temporal SUVmean. *Statistically significant difference. Amyg SUVmax and AmygA were analyzed with Mann-Whitney *U* test. Temporal SUVmean was analyzed with Student’s *t*-test.

We first investigated whether the AmygA was increased in postmenopausal women with osteoporosis. **Figure 2A** presents a quantitative comparison of PET/CT images that highlights the characteristic tracer uptakes at the target regions between the patients with- and without osteoporosis. As shown in **Figure 2**, the osteoporosis group showed significantly higher Amyg SUVmax (3.85 ± 0.82 *vs*. 2.89 ± 0.9, *p* < 0.001, **Figure 2B**) and AmygA (0.81 ± 0.16 *vs*. 0.61 ± 0.13, *p* < 0.001, **Figure 2D**) than the women without osteoporosis. However, Temporal SUVmean showed no significant difference between the groups (*p* = 0.69, **Figure 2C**). In further correlation analysis, both Amyg SUVmax and AmygA showed significant negative low correlation with both LS- and FN BMD T-scores (**Table 2**). Interestingly, unlike to Amyg SUVmax, only AmygA was significantly positively correlated with the level of hsCRP, which is a surrogate marker of systemic inflammation.

**Table 2:** Correlation analysis.

	Amyg SUVmax	Temporal SUVmean	AmygA
LS BMD T-score^†^	-0.321**	-0.084	-0.343***
FN BMD T-score^†^	-0.147*	-0.033	-0.185*
hsCRP	0.179	0.038	0.198*

Amyg SUVmax, maximum standardized uptake value of amygdala; Temporal SUVmean, mean standardized uptake value of temporal lobe; AmygA, the metabolic activity of amygdala; Amyg, SUVmax/Temporal SUVmean; LS BMD, lumbar spine bone mineral density; FN BMD, femoral neck bone; hsCRP, high-sensitivity C-reactive protein. ^†^Partial correlation analysis was performed after adjustment of age, BMI, WC, PWI-SF score, postmenopausal period, and hsCRP.

*p < 0.05, **p < 0.01, ***p < 0.001.

### AmygA Can Predict Osteoporosis in Postmenopausal Women

**Figure 3:**
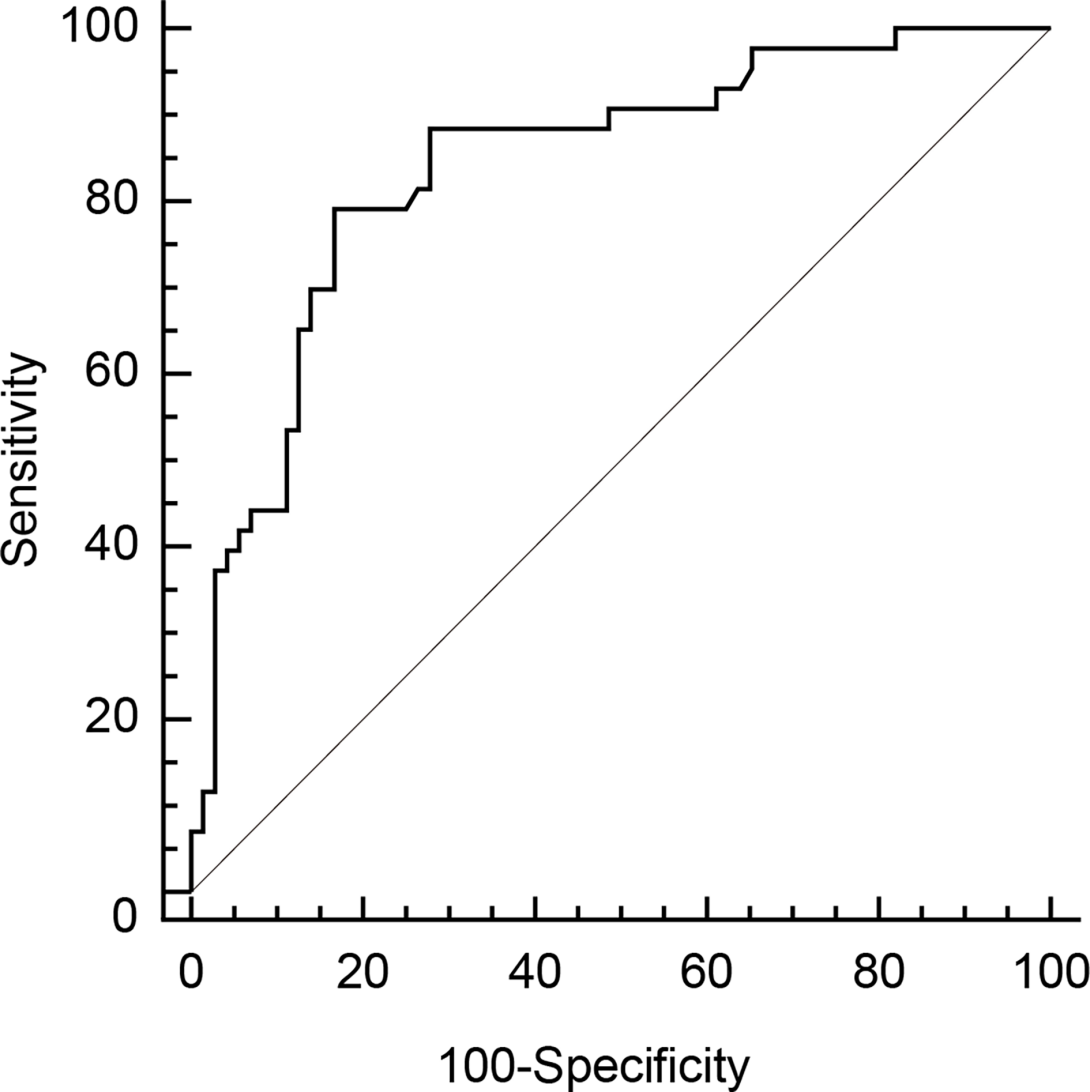
Receiver operating characteristic (ROC) curve analysis of AmygA to identify osteoporosis in postmenopausal women. The optimal cut-off value was chosen by the maximum Youden index [sensitivity−(1−specificity)] on the ROC curve.

We performed ROC curve analysis to explore the optimal cut-off AmygA value to identify osteoporosis. As shown in **Figure 3**, the optimal cut-off AmygA value for prediction of osteoporosis was 0.69 with a sensitivity of 79.1% and a specificity of 83.3%. The corresponding area under the curve (AUC) was 0.841 (95% confidence interval 0.762 – 0.903; standard error 0.0387; *p* < 0.001).

### AmygA Is Independently Associated With Osteoporosis

Next, using the optimal cut-off AmygA value, we performed univariate- and multivariate logistic regression analyses to investigate the association between AmygA and osteoporosis. As shown in **Table 3**, univariate logistic regression analysis exhibited that WC, postmenopausal period, and AmygA were significantly associated with osteoporosis. After adjustment of age, BMI, alcohol-, smoking-, and exercise habit, subsequent multivariate analysis showed that both postmenopausal period and AmygA were independently associated osteoporosis. In addition, among the included variables, AmygA showed the highest odds ratio for osteoporosis.

**Table 3:** Uni- and multivariate logistic regression analyses for the associated predictors of osteoporosis in postmenopausal women.

Variables	Univariate analysis		Multivariate analysis	
	OR (95% CI)	*p*	OR (95% CI)	*p*
Age (Continuous)	1.045 (0.981–1.112)	0.175		
WC (Continuous)	1.055 (1.002–1.111)	0.043*	1.049 (0.98–1.122)	0.168
BMI (Continuous)	1.096 (0.975–1.233)	0.126		
Alcohol frequency (None *vs* Light and Heavy)	0.542 (0.243–1.205)	0.133		
Alcohol amount (None *vs* Light and Heavy)	0.603 (0.274–1.329)	0.21		
Smoking (Never *vs* Ex and Current)	0.696 (0.17–2.849)	0.615		
Regular exercise (No *vs* Yes)	0.882 (0.322–2.418)	0.808		
Postmenopausal period (≤5 years *vs* >5 years)	3.38 (1.419–8.054)	0.006*	2.951 (1.013–8.6)	0.047*
AmygA (≤0.69 *vs* >0.69)	18.889 (7.224–49.393)	<0.001*	17.378 (6.426–47)	<0.001*

OR, odds ratio; CI, confidence interval; WC, waist circumference; BMI, body mass index; AmygA, the metabolic activity of amygdala.

*Statistically significant difference.

### AmygA Reflects the Level of Psychological Stress

**Figure 4:**
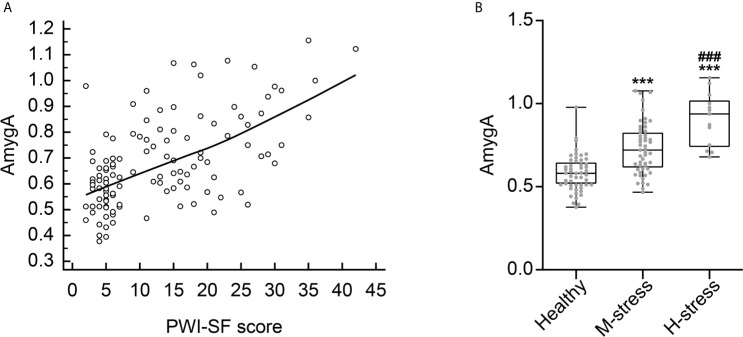
**(A)** Correlation scatter plot between AmygA and PWI-SF score. **(B)** Comparison of AmygA according to the severity of psychological stress. Healthy, *n* = 51; M-stress, *n* = 51; H-stress, *n* = 13. PWI-SF; psychosocial well-being index-short form, Healthy, healthy group; M-stress, moderate stress risk group; H-stress, high stress risk group. ***p < 0.001; *vs*. Healthy, ^###^ *p* < 0.001; *vs*. M-stress. AmygA was analyzed with one-way analysis of variance (ANOVA) with *post-hoc* Tukey’s test.

We also examined whether the AmygA was associated with the level of psychological stress. As shown in **Figure 4A**, AmygA was significantly positively correlated with the PWI-SF score (*r* = 0.541, *p* < 0.001). Notably, AmygA was highest in participants with high risk of stress, intermediate in participants with moderate risk of stress, and lowest in healthy group (0.91 ± 0.16 *vs*. 0.73 ± 0.16 *vs*. 0.58 ± 0.11, *p* < 0.001, respectively) (**Figure 4B**). Thus, AmygA could reflect the level of psychological stress.

### AmygA Is Associated With Osteoporosis in Participants With Moderate- and High Psychological Stress

Next, we performed univariate- and multivariate logistic regression analyses to evaluate the association between AmygA and osteoporosis in both healthy group and patients with psychological stress. In healthy group, as shown in **Table 4**, AmygA showed no significant association with osteoporosis. In contrast, in participants with moderate- and high psychological stress, AmygA was significantly associated with osteoporosis (**Tables 5** and **6**). Furthermore, the odds ratio of AmygA for osteoporosis was higher in participants with high psychological stress than that in participants with moderate psychological stress.

**Table 4:** Uni- and multivariate logistic regression analyses for the associated predictors of osteoporosis in postmenopausal women without psychological stress.

Variables	Univariate analysis		Multivariate analysis	
	OR (95% CI)	*p*	OR (95% CI)	*p*
Age (Continuous)	0.554 (0.937–1.13)	0.554		
WC (Continuous)	1.094 (1.002–1.111)	0.041*	1.073 (0.981–1.174)	0.125
BMI (Continuous)	1.142 (0.965–1.352)	0.123		
Alcohol frequency (None *vs* Light and Heavy)	0.346 (0.064–1.865)	0.217		
Alcohol amount (None *vs* Light and Heavy)	0.346 (0.064–1.865)	0.217		
Smoking (Never *vs* Ex and Current)	2.5 (0.202–30.988)	0.476		
Regular exercise (No *vs* Yes)	0.458 (0.05–4.16)	0.488		
Postmenopausal period (≤5 years *vs* >5 years)	5.44 (0.622–47.561)	0.126		
AmygA (≤0.69 *vs* >0.69)	8 (0.918–69.721)	0.06	5.736 (0.624–52.688)	0.123

Without psychological stress was defined as the healthy group.

OR, odds ratio; CI, confidence interval; WC, waist circumference; BMI, body mass index; AmygA, the metabolic activity of amygdala.

*Statistically significant difference.

**Table 5:** Uni- and multivariate logistic regression analyses for the associated predictors of osteoporosis in postmenopausal women with moderate risk of stress.

Variables	Univariate analysis		Multivariate analysis	
	OR (95% CI)	*p*	OR (95% CI)	*p*
Age (Continuous)	1.117 (0.986–1.266)	0.082		
WC (Continuous)	1.028 (0.95–1.113)	0.488		
BMI (Continuous)	1.065 (0.877–1.293)	0.524		
Alcohol frequency (None *vs* Light and Heavy)	0.778 (0.256–2.363)	0.658		
Alcohol amount (None *vs* Light and Heavy)	0.778 (0.256–2.363)	0.658		
Smoking (Never *vs* Ex and Current)	0.478 (0.079–2.879)	0.421		
Regular exercise (No *vs* Yes)	3.789 (0.686–20.946)	0.127		
Postmenopausal period (≤5 years *vs* >5 years)	4.318 (1.296–14.383)	0.017*	3.196 (0.837–12.201)	0.089
AmygA (≤0.69 *vs* >0.69)	9 (2.487–32.567)	0.001*	7.533 (2–28.37)	0.003*

OR, odds ratio; CI, confidence interval; WC, waist circumference; BMI, body mass index; AmygA, the metabolic activity of amygdala.

*Statistically significant difference.

**Table 6:** Uni- and multivariate logistic regression analyses for the associated predictors of osteoporosis in postmenopausal women with high risk of stress.

Variables	Univariate analysis		Multivariate analysis	
	OR (95% CI)	*p*	OR (95% CI)	*p*
Age (Continuous)	1.064 (0.778–1.457)	0.697		
WC (Continuous)	1.026 (0.829–1.269)	0.814		
BMI (Continuous)	1.011 (0.659–1.551)	0.96		
Alcohol frequency (None *vs* Light and Heavy)	0.375 (0.017–8.103)	0.532		
Alcohol amount (None *vs* Light and Heavy)	0.857 (0.055–13.479)	0.913		
Smoking (Never *vs* Ex and Current)	0	1		
Regular exercise (No *vs* Yes)	0	0.999		
Postmenopausal period (≤5 years *vs* >5 years)	3.5 (0.284–43.161)	0.328		
AmygA (≤0.69 *vs* >0.69)	24 (1.111–518.581)	0.043*	24 (1.111–518.581)	0.043*

OR, odds ratio; CI, confidence interval; WC, waist circumference; BMI, body mass index; AmygA, the metabolic activity of amygdala.

*Statistically significant difference.

## Discussion

To the best of our knowledge, this is the first study to demonstrate the relationship between stress-related brain neural activity and osteoporosis as assessed by ^18^F-FDG PET/CT in postmenopausal women. In this study, we clearly revealed that the metabolic activity of amygdala defined as AmygA and evaluated by ^18^F-FDG PET/CT was associated with osteoporosis and could reflect the levels of psychological stress in postmenopausal women.

The present study highlights the function of amygdala as a key brain neural component in the mechanism that physiological and emotional response to psychological stress, thereby promoting osteoporosis in postmenopausal women. Previous studies have reported that psychological stress prompts the amygdala’s projections to the brainstem thereby triggering the activation of HPA axis and sympathetic nervous system, which collectively lead to increases in circulating glucocorticoids and catecholamines (5, 6, 8). Elevated levels of glucocorticoids can inhibit osteoblast differentiation and proliferation thereby affecting bone formation and resorption whereas increased catecholamines can increase bone resorption through promotion of osteoclastogenesis, which synergistically affect bone remodeling process thereby resulting in the reduction of bone mass (6, 15–17). Notably, in this study, there was a stepwise increase in associations between AmygA and osteoporosis from the healthy group to the moderate risk of stress- and the high risk of stress group. Thus, higher stress levels might elevate the metabolic activity of amygdala proportionally thereby boosting sending out signals to increase stress hormones that could induce an imbalance in favor of bone resorption over formation, which leads to osteoporosis. 

Furthermore, although the detailed underling mechanism remains unclear, previous clinical and animal studies have reported that estrogen might contribute to the normal physiologic function of the HPA axis response and lower endogenous estrogen levels could augment the HPA axis response to psychological stress (18, 19). Interestingly, amygdala, that have connections to HPA, is considered as a brain target for estrogen’s effect on psychological stress (18, 20) and recently, Estrada et al. (21) report that direct estrogen injection into the amygdala attenuates emotional stress responses in ovariectomized rats. Thus, decreased estrogen levels, as occur with postmenopausal women, can worsen the psychological stress-mediated osteoporosis which are also consistent with our results. Furthermore, our findings provide an important physiological relationship between the amygdala and psychological stress-mediated osteoporosis in postmenopausal women.

In the present study, we found that hsCRP, a surrogate marker for systemic inflammation, was significantly correlated with AmygA and was increased in postmenopausal women with osteoporosis. Chronic psychological stress has been known to be associated with elevated systemic inflammation (22, 23) and Tawakol et al. (8) found that AmygA is correlated with psychological stress and CRP, which were consistent with our results. Thus, AmygA might reflect the inflammatory burden of psychological stress which could be related with osteoporosis. Interestingly, patients with chronic inflammatory diseases have a higher incidence of osteoporotic fractures (24) and high levels of pro-inflammatory cytokines such as interleukin-6 (IL-6) and tumor necrosis factor alpha (TNF-α) inhibit osteoblast function and promote osteoclast function, thereby having a detrimental effect on osteoporosis (1, 25). However, conversely, it has been also suggested that increased inflammation factors might inhibit osteoclast activity thereby improving osteoporosis (26). Thus, the roles of systemic inflammation in psychological stress and in osteoporosis seem highly complex and further studies are warranted to explore the relationship between AmygA, systemic inflammation, and bone health.

A growing body of evidence suggests that targeting psychological stress is a promising therapeutic strategy against osteoporosis (27). In postmenopausal women, pharmacologic interventions such as strontium ranelate and beta-blockers might improve mental health which could lead to have a beneficial effect on osteoporosis (28, 29). Furthermore, life style modification and dietary supplements such as exercise and calcium and vitamin D could also impact metal health thereby improving bone health (27, 30, 31). Given that amygdala is regarded as a key neural component of stress response system, which contributes to the increased risk of osteoporosis, it is conceivable that AmygA evaluated by ^18^F-FDG PET/CT could be used as a potential surrogate marker of psychological stress levels, thereby assessing the therapeutic effect against psychological stress and its related osteoporotic bone change in postmenopausal women.

Our study has several limitations. First, because of the cross-sectional study design, we could not find causality or relation directionality. Further longitudinal large study is needed to validate our findings. Second, due to the limited resolution of clinical ^18^F-FDG PET/CT imaging, we were unable to evaluate other brain regions involved in stress-responsive neural circuit such as the hippocampus and prefrontal cortex. Third, we could not directly evaluate the markers of the HPA axis and sympathetic nervous system activity. Fourth, we could not control all the health behaviors and nutritional factors such as caffeine consumption, dietary calcium intake, or vitamin D supplementation that might potentially affect the osteoporosis in postmenopausal women. Fifth, although AmygA is known to relatively stable over time and associates with a human’s perceived stress (8, 9, 32), there is a possibility that it can change with time. A further prospective study is needed to determine the optimal imaging time interval to reflect the levels of individual’s perceived stress and its adverse pathologic consequence. Sixth, the levels of psychological stress can also change with time. However, we evaluate the levels of psychological stress at one time before taking ^18^F-FDG PET/CT. The effect of changes in the level of psychological stress on osteoporosis should be addressed in further studies. Seventh, BMD T-score itself showed weak but significant correlation with both Amyg SUVmax and AmygA. However, when we applied the diagnostic criteria of osteoporosis (BMD T-score ≤ -2.5), AmygA was significantly associated with participants with osteoporosis. Considering the possible causal relationship between stress-related amygdala activity and osteoporosis, we thought this discrepancy might result from the different response speed of amygdala and bone microarchitecture to the levels of psychological stress. Further studies are needed to elucidate the pathophysiological process of amygdala acting on bone in the pathogenesis of osteoporosis. Eighth, we only used hsCRP as a systemic inflammation surrogate marker to correlate with AmygA. We did not assess other markers of systemic inflammation such as IL-6 or TNF-α. Further research is needed to explore the intrinsic relationship between AmygA and systemic inflammation. Finally, although this study was performed on postmenopausal women who were expected to have lower levels of estrogen, we could not directly measure the level of estrogen in each participant. As amygdala is an action site of estrogen’s effect on stress, further study is also warranted to investigate the relationship between the level of estrogen and AmygA. Nevertheless, the findings of our study counterbalanced these limitations by using a unique non-invasive functional imaging for the quantification of the metabolic activity of amygdala, to explore the relationship between stress-related brain neural activity and osteoporosis in postmenopausal women.

In conclusion, we provide strong evidence that the metabolic activity of amygdala, defined as AmygA and assessed by ^18^F-FDG PET/CT was associated with the level of psychological stress and osteoporosis in postmenopausal women. These findings could support the proposed mechanistic relationship between stress-related amygdala metabolic activity and osteoporosis in postmenopausal women. Moreover, this study further highlights the potential role of AmygA as a useful surrogate marker for reflecting psychological stress level, when assessing postmenopausal women at risk for osteoporosis.

## Data Availability Statement

The original contributions presented in the study are included in the article/supplementary material. Further inquiries can be directed to the corresponding author.

## Ethics Statement

The studies involving human participants were reviewed and approved by The Institutional Review Board of Korea University Anam Hospital. Written informed consent for participation was not required for this study in accordance with the national legislation and the institutional requirements.

## Author Contributions

Conceptualization: KP and SK. Data curation: KP, HK, and CJ. Formal analysis: KP, HK, and CJ. Investigation: KP and HK. Methodology: KP, HK, and CJ. Project administration: KP and SK. Validation: KP and HK. Visualization: KP and CJ. Writing-original draft: KP. Writing-review and editing: SK. Supervision: SK. Funding acquisition: SK. All authors contributed to the article and approved the submitted version.

## Funding

This work was funded by Korea University Anam Hospital, grant numbers K2102671 and K2107351. The authors declare that this study received funding from KOREA HYDRO & NUCLEAR POWER CO., LTD (20-Tech-15). The funder was not involved in the study design, collection, analysis, interpretation of data, the writing of this article or the decision to submit it for publication.

## Conflict of Interest

The authors declare that the research was conducted in the absence of any commercial or financial relationships that could be construed as a potential conflict of interest.

## Publisher’s Note

All claims expressed in this article are solely those of the authors and do not necessarily represent those of their affiliated organizations, or those of the publisher, the editors and the reviewers. Any product that may be evaluated in this article, or claim that may be made by its manufacturer, is not guaranteed or endorsed by the publisher.
